# Mitochondrial-Derived Peptides Are Down Regulated in Diabetes Subjects

**DOI:** 10.3389/fendo.2019.00331

**Published:** 2019-05-31

**Authors:** Manjunath Ramanjaneya, Ilham Bettahi, Jayakumar Jerobin, Prem Chandra, Charbel Abi Khalil, Monica Skarulis, Stephen Lawrence Atkin, Abdul-Badi Abou-Samra

**Affiliations:** ^1^Hamad Medical Corporation, Department of Medicine, Qatar Metabolic Institute and Interim Translational Research Institute, Academic Health System, Doha, Qatar; ^2^Epigenetics Cardiovascular Laboratory, Department of Genetic Medicine, Weill Cornell Medicine Qatar, Doha, Qatar; ^3^Research Department, Weill Cornell Medicine Qatar, Doha, Qatar

**Keywords:** type 2 diabetes, mitochondrial derived peptides, humanin, mitochondrial open reading frame of the 12S rRNA type-c, glycated hemoglobin, triglycerides and insulin resistance

## Abstract

**Background:** Mitochondrial dysfunction is implicated in the pathogenesis of Type 2 diabetes (T2D) and the development of diabetes related complications such as cardiovascular disease and stroke. Mitochondria produce several small polypeptides that may influence mitochondrial function and may impact on insulin sensitivity, such as humanin (HN) and the mitochondrial open reading frame of the 12S rRNA type-c (MOTS-c) that are mitochondrial derived proteins (MDP). The aim of this study was to determine MDP in normal, prediabetes and diabetes subjects.

**Subjects and Measurements:** In this cross-sectional study, we analyzed the serum concentrations of MDP and adiponectin (ADP) in 225 subjects: normal (*n* = 68), pre-diabetes (*n* = 33), T2D less than (good control; *n* = 31), and greater than HbA1c 7% (poor control; *n* = 93) subjects. The relationship of serum MDP and ADP concentrations with biochemical and anthropometric measurements were performed and assessed by multilinear regression.

**Results:** Serum HN concentrations were lower in T2D (*p* < 0.0001) and negatively correlated with age (*p* < 0.0001), HbA1c (*p* < 0.0001), glucose (*p* < 0.0001), triglycerides (*p* < 0.003), ALT (*p* < 0.004), and TG/HDL ratio (*p* < 0.001). Circulating HN levels were positively correlated to cholesterol (*p* < 0.017), LDL (*p* < 0.001), and HDL (*p* < 0.001). Linear regression analysis showed that HbA1c and ALT were two independent predictors of circulating HN. Similarly, serum MOTS-c was significantly lower in T2D subjects compared to controls (*p* < 0.007). Circulating MOTS-c positively correlated with BMI (*p* < 0.035), total cholesterol (*p* < 0.0001), and LDL (*p* < 0.001) and negatively correlated with age (*p* < 0.002), HbA1c (*p* < 0.001), and glucose (*p* < 0.002). Serum ADP concentrations were lower in T2D (*p* < 0.002) and negatively correlated with HbA1c (*p* < 0.001), weight (*p* < 0.032) TG (*p* < 0.0001), and ALT (*p* < 0.0001); and positively correlated with HDL (*p* < 0.0001) and HN (*p* < 0.003). Linear regression analysis showed that HbA1c and weight were two independent predictors of circulating ADP. Multilinear regression showed that HN and MOT-c correlated with each other, and only HN correlated with HbA1c.

**Conclusion:** The MDPs HN and MOT-c, similar to ADP, are decreased in T2D and correlate with HbA1c. The data provide an additional evidence that mitochondrial dysfunction contributes to glycemic dysregulation and metabolic defects in T2D.

## Introduction

Type 2 diabetes (T2D) and its associated comorbidities and complications are global health problems especially for developing nations (1), and in Qatar, 17–20% of adults are estimated to be diabetic (2). This is a significant risk factor for developing major life-threatening illness, including cardiovascular disease, stroke, chronic kidney disease (3), and cancer, which are linked with a markedly diminished life expectancy (4–12). T2D is characterized by insulin resistance (IR) through which insulin target tissue is unable to respond normally to insulin reflecting insulin deficiency (13). Mitochondria are central for maintaining metabolic health and cellular energy homeostasis, and mitochondrial dysfunction leads to inefficiency in the electron transport chain and beta-oxidation, thus trigging IR (14–16). It has been suggested that mitochondrial dysfunction is associated with T2D as well as in diabetes related complications (17–19). Blake et al. demonstrated that mitochondrial dysfunction was involved in diabetes-induced complications affecting the kidneys, nervous system, heart and retina (19), and it was suggested that mitochondrial dysfunction-induced oxidative stress was contributory for these complications (19). Others reported that mitochondrial oxidative stress was responsible for hyperglycemia-induced disorders affecting blood vessels, kidney, pancreatic β cells, and liver (20).

Mitochondria produce numerous small polypeptides from their short open reading frame (sORF) regions of mtDNA that have significant biological activity (21). These include humanin, six small-humanin like peptides, and MOTS-c (mitochondrial open reading frame of the 12S rRNA type-c), together termed mitochondrial derived peptides (MDP). Studies in rodents demonstrate that MDP promote mitochondrial metabolism and regulate critical processes such as aging, inflammation, and reverse insulin resistance (22–25). Humanin (HN) is a 24-amino acid neuro-protective polypeptide discovered in healthy neurons of Alzheimer's patients. HN and HN-analogs regulate important cellular function such as promoting cell survival, metabolism, response to stressors, and inflammation both *in vivo* and *in vitro* (23). MOTS-c inhibits folate cycle, purine biosynthesis and promotes 5′ AMP-activated protein kinase (AMPK) activation and mitochondrial metabolism. (24). The mitochondrial derived peptides (MDP) humanin and MOTSc are key regulators of insulin sensitivity and metabolism. The insulin sensitizing properties of MPDs have been demonstrated previously in cellular and rodent models. Administration of HN and HN-GF6A reduced blood glucose and promoted insulin sensitivity in Zuker diabetic rats, whilst in the non-obese diabetic mouse model, HN administration prevented the early onset of type 1 diabetes (26). Similar to HN, short humanin like peptide 2 (SHLP2) decreased hepatic glucose production and increased glucose disposal into peripheral tissue in hyperinsulinemic-euglycemic clamp studies in mice (27). The role of MDP in insulin sensitivity was supported further with the discovery of MOTS-c. Overexpression of MOTS-c increased glucose uptake in myoblasts and intraperitoneal administration of MOTS-c activated AMPK pathway and GLUT4 expression in skeletal muscle, and reversed age and diet induced IR in mice (24). This data suggested that, in rodent models, MDP had a role in improving T2D induced insulin resistance. However, the association of MDP in obese and T2D subjects with insulin resistance in human is not clearly understood.

Adipokines are a family of cytokines (leptin, adiponectin, resistin, and visfatin) that are released mainly by activated macrophages of adipocytes. They play an essential role in the development of insulin resistance and T2D, and are associated with an increased risk of obesity related cardiovascular disease (28, 29). It has been suggested that adiponectin may influence mitochondrial biogenesis and hence MDP production (30). Therefore, we sought to investigate the association of serum MDP in normal, prediabetes and T2D subjects to gain insight as to their role in diabetes and to determine if they were related to adiponectin levels.

## Materials and Methods

This was a cross-sectional study performed using blood samples collected from Qatari T2D subjects attending the outpatient department and endocrinology clinics at Hamad Medical Corporation according to an IRB approved protocol [Hamad Medical Corporation (HMC#9093/09) and Weill Cornell Medicine Qatar (WCM-Q#13-00063)]. All study participants gave informed written consent before participation in the study.

### Study Participants

Two hundred and twenty five Qatari subjects, aged 30–75 years, were enrolled in a population-based cross-sectional study. The T2D subjects were recruited from the outpatient department at Hamad Medical Corporation, and healthy controls were either family members or visitors in the waiting area of the outpatient clinics. Pregnant women, subjects with type 1 diabetes, secondary diabetes, chronic hematological disorders known to affect HbA1c levels, active malignancy during the past 5 years or other chronic conditions not related to diabetes and its complications were excluded.

SThe study subjects were divided into 4 groups based on their HbA1c following NICE criteria for prediabetes/diabetes diagnosis. The study group consisted of 68 healthy volunteer control subjects (HbA1c < 6%), 33 volunteers with prediabetes (HbA1c 6.0–6.4%) and 124 T2D (31 T2D (HbA1c < 7.0%, and 93 T2D HbA1c > 7%). Subjects with T2D were included within 10 years of diagnosis and free of complications. None of the prediabetes subjects were on an insulin sensitizer. The age, sex, height, weight, body mass index (BMI), random plasma glucose, glycated hemoglobin (HbA1c), total cholesterol (TCH), triglycerides, high-density lipoproteins cholesterol *(*HDL), low-density lipoprotein cholesterol (LDL), alanine aminotransferase (ALT), systolic blood pressure, and diastolic blood pressure were recorded at the time of participation in the study. Serum humanin (HN) concentrations were measured using a commercially available ELISA kit (MyBioSource, CA, USA; Catalog number: MBS914849) according to manufacturer's recommended protocol, with a detection range between 28 and 1,800 pg/ml and intra-assay coefficient of variation of <8% and inter-assay coefficient of variation of <10%. Serum MOTS-c concentrations were measured using a commercially available extraction-free EIA kit (Peninsula Laboratories International, CA, USA: Catalog number: S-1526) according to manufacturer's recommended protocol, with a detection range between 0.10 and 100 ng/ml. Serum ADP concentrations were measured using a commercially available Bio-Plex Pro^™^ human diabetes adiponectin immune assay kit (Bio-Rad Laboratories Ltd, Hertfordshire, U.K, Catalog number # 171a7003m) according to the manufacturer's recommended protocol, with a detection range between 0.17 and 704.02 ng/ml) and intra-assay coefficient of variation of <4% and inter-assay coefficient of variation of <3%.

### Statistical Analysis

Data points are represented as means ± SD or median and interquartile range unless otherwise stated. The differences in mean serum MDP concentration between control, prediabetes T2D good control and in T2D poor control subjects were determined by one-way ANOVA followed by *post-hoc* tests with Bonferroni correction adjusting for alpha-error in the multiple group comparison. Spearman rank correlation was used to assess the relationship between serum MDP with clinical and biochemical parameters. Multiple linear regression analysis was conducted using individual covariant found to be significant in Spearman correlation analysis using SPSS software version 22.0 (SPSS, Inc, Chicago, IL, USA) and GraphPad Prism version 5. Assumptions of linear regression such as linearity, multivariate normality, multicollinearity, and homoscedasticity were tested and found to be satisfactory. The *P* < 0.05 was considered significant.

## Results

### Anthropometric and Biochemical Variations in Control and T2D Subjects

The study group demographics, anthropometrics and biochemical data are shown in Table 1A. The control group was slightly younger than the groups with pre-diabetes and diabetes. However, there was no difference in BMI across the groups. Not unexpectedly and in keeping with the groups defined by HbA1c, there were differences observed in triglycerides and HDL (Table 1A). However, the groups were generally similar with minor differences.

**Table 1A:** Clinical and metabolic features of study subjects.

	**Control (*n* = 68)**	**Prediabetes (*n* = 33)**	**T2D (HbA1c <7%) (*n* = 31)**	**T2D (HbA1c >7%) (*n* = 93)**	**ANOVA**
	**Mean ± SD (Interquartile range)**	**Mean ± SD (Interquartile range)**	**Mean ± SD (Interquartile range)**	**Mean ± SD (Interquartile range)**	***P***
Age (year)	49.3 ± 10.6 (41.0–55.8)	54.1 ± 9.6 (47.0–60.5)	54.7 ± 11.2 (48.0–63.0)	57.2 ± 8.3 (51.0–63.0)	<0.0001
Sex (men/women)	33/35	17/16	13/18	56/37	NS
Height (m)	1.6 ±0.1 (1.6–1.7)	1.7 ± 0.1 (1.6–1.8)	1.6 ± 0.1 (1.6–1.7)	1.6 ± 0.1 (1.6–1.7)	0.373
Weight (kg)	95.9 ± 17.5 (85.0–106.8)	96.4 ± 20.3 (81.5–107.5)	91.2 ± 18.0 (75.0–103.0)	90.1 ±16.5 (77.0–101.9)	0.109
BMI (Kg/m^2^)	35.2 ± 5.6 (31.2–39.1)	34.6 ± 5.8 (30.2–38.7)	34.5 ± 6.3 (30.7–40.1)	33.5 ±5.8 (29.2–37.3)	0.32
SBP (mm Hg)	125.8 ± 14.5 (114.3–135.3)	134.8 ± 22.0 (116.0–155.3)	145.9 ± 21.8 (128.0–151.0)	139.4 ± 21.0 (122.8–153.5)	0.016
DBP (mm Hg)	74.3 ±10.7 (67.5–80.0)	73.3 ± 16.5 (60.8–85.0)	76.9 ± 9.7 (70.0–87.0)	76.2 ± 11.1 (68.5–82.3)	0.785
HbA1C %	5.4 ±0.4 (5.1–5.8)	6.2 ± 0.1 (6.1–6.3)	6.6 ±0.2 (6.4–6.7)	9.0 ±1.6 (7.6–10.0)	<0.0001
TCH (mmol/L)	4.9 ±1.1 (4.3–5.7)	4.4 ± 0.7 (3.9–4.9)	4.8 ± 1.0 (3.8–5.6)	4.7 ±1.3 (3.7–5.6)	0.252
TG (mmol/L)	1.3 ±0.6 (0.9–1.5)	1.4 ± 0.8 (1.0–1.6)	1.4 ± 0.9 (0.9–1.7)	2.1 ± 1.4 (1.1–2.8)	<0.0001
HDL (mmol/L)	1.3 ± 0.3 (1.1–1.5)	1.2 ± 0.3 (0.9–1.4)	1.3 ± 0.4 (1.0–1.6)	1.1 ± 0.3 (0.9–1.3)	0.003
LDL (mmol/L)	3.1 ± 1.0 (2.4–3.6)	2.6 ± 0.6 (2.2–3.1)	2.8 ± 1.0 (2.1–3.5)	2.7 ± 1.1 (1.9–3.3)	0.072
ALT (U/L)	29.0 ± 25.7 (16.0–34.8)	33.9 ± 29.9 (16.0–47.5)	26.6 ± 14.2 (15.0–32.0)	30.7 ± 17.1 (18.0–39.0)	0.572
TG/HDL	1.1 ± 0.7 (0.6–1.3)	1.3 ± 0.9 (0.7–1.7)	1.3 ± 1.2 (0.6–1.9)	2.1 ± 1.7 (1.1–2.5)	<0.0001
HN (pg/ml)	1292.8± 56.4 (1028.1–1595.1)	783.7 ± 620.2 (252.6–1128.7)	565.3 ± 402.9 (289.4–667.6)	372.5 ± 275.5 (193.9–457.6)	<0.0001
MOTS-c (pg/ml)	235.3 ±181.6 (146.2–252.9)	154.3 ± 66.3 (110.9–200.7)	186.3 ±125.6 (105.1–214.2)	157.7 ± 136.6 (53.7–214.6)	0.007
ADP (μg/ml)	10.4 ± 7.8 (5.8–12.4)	7.0 ± 5.1 (3.5–8.2)	7.4 ± 4.3 (3.8–11.3)	6.6 ± 4.6 (3.1–8.4)	0.002

*Data are presented as Mean ± SD (Interquartile range). Comparision between the groups were done by one-way ANOVA. MOTS-c, mitochondrial open reading frame of the 12S rRNA type-c; HN, humanin; ADP, adiponectin; BMI, body mass index; SBP, systolic blood pressure; DBP, diastolic blood pressure; TCH, total cholesterol; HDL, high density lipoprotein; LDL, low density lipoprotein; TG, triglycerides; HbA1c, glycated hemoglobin; ALT, alanine aminotransferase; TCH, total cholesterol; P < 0.05 was considered as significant*.

### HbA1c and Serum HN, MOTS-c, and ADP Levels

**Figure 1:**
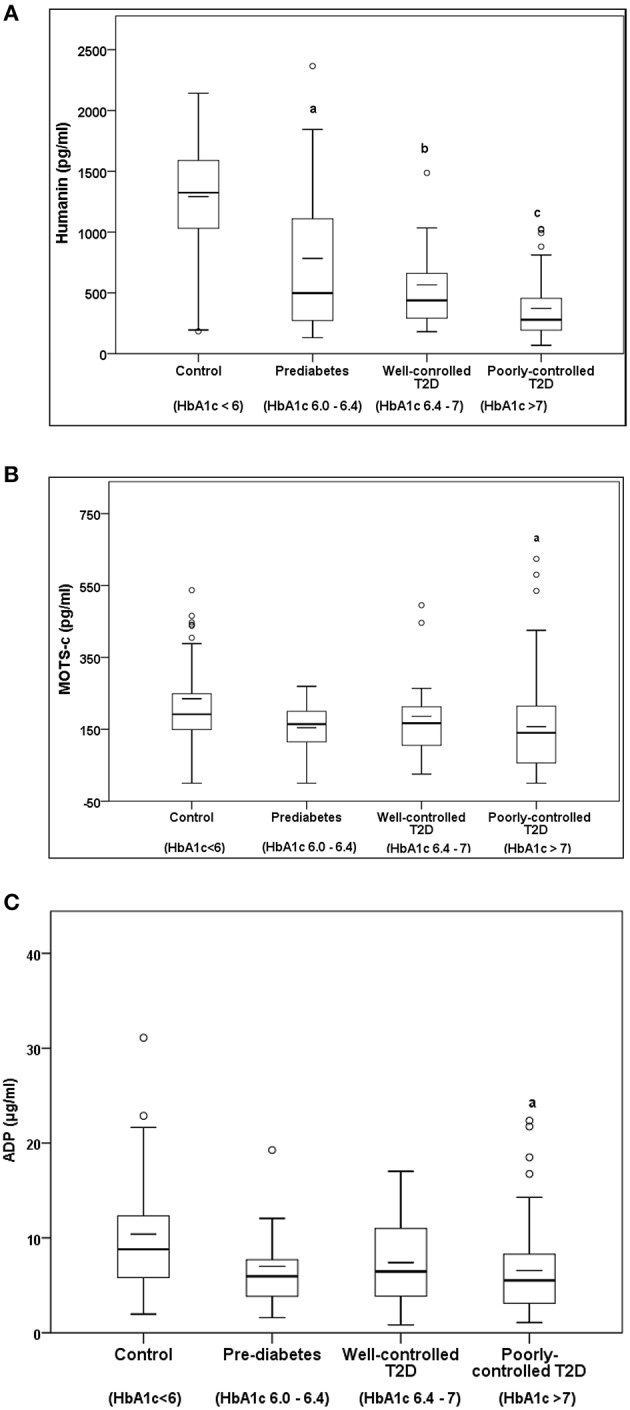
The Boxplot of **(A)** HN, **(B)** MOTS-c, and **(C)** ADP concentration in circulation and its association with variation of HbA1C in control, prediabetes, well-controlled T2D and poorly-controlled T2D subjects. Lower border of the box plots represent the 25th percentile and the upper border represents the 75th percentile ± SD. Short–inside the Boxplot represents statistical mean for HN **(A)**, MOTS-c **(B)**, and ADP **(C)**, respectively. Alphabets on the Boxplot represent significant difference between groups. (a) *P* < 0.0001 between control and prediabetes, (b) *p* < 0.0001 between control and T2D good control, and (c) *p* < 0.0001 between control and T2D poor control. Out layers are highlighted as circles.

Serum HN levels differed according to level of glycemia (*p* < 0.0001) (Table 1A and Figure 1A). Compared to controls, there was a significant decrease in HN in prediabetes, the T2D good control and the T2D poor control groups. Further multiple comparison test with Bonferroni correction showed that HN levels were lower but not significantly different in T2D compared to prediabetes, and there was further decrease in poorly controlled T2D compared to prediabetes (*p* < 0.0001). Patients with well-controlled and poorly controlled T2D have similar HN levels (Table 1B). Serum MOTS-c levels were significantly lower in poorly controlled T2D patients compared to healthy controls (Table 1A and Figure 1B). No impact of the type of antidiabetic treatment including metformin on HN or MOTS-c levels was observed (Table 3). There were no significant differences in MDP between male and female subjects in the four groups. The mean and SD for HN in combined group was (781.4 ± 580.0 pg/ml) in male and (798.8 ± 609.1 pg/ml) in female and the MOTS-c levels were (209.8 ± 132 pg/ml) in male and (221.6 ± 180 pg/ml) in female subjects. The relationship between circulating HN, MOTS-c, and ADP with HbA1c are shown in Figure 1. As the HbA1c levels increased serum HN and MOTS-c levels decreased. Control subjects had higher HN and MOTS-c levels compared to subjects with prediabetes, well-controlled T2D or poorly-controlled T2D groups, respectively (Figure 1, Table 1A). One way ANOVA analysis showed that mean serum ADP was significantly lower in T2D (*p* < 0.002) compared to controls (Table 1A and Figure 1C). Males (6.8 ± 4.3 μg/ml) had lower ADP compared to females (9.4 ± 7.2 μg/ml, *p* < 0.002). The relationship between circulating ADP and HbA1c are shown in Figure 1C. Control subjects had higher ADP levels compared to subjects with prediabetes, well-controlled T2D or poorly-controlled T2D groups, respectively (Figure 1C, Table 1A).

**Table 1B:** *Post-hoc* analysis with bonferroni correction adjusting for alpha error.

		**HN**	**MOTS-c**	**ADP**
		***p***	***p***	***p***
Control	Pre-diabetes	<0.0001	0.07	0.098
	Well-controlled T2D	<0.0001	0.777	0.184
	Poorly-controlled T2D	<0.0001	0.008	0.001
Pre-diabetes	Control	<0.0001	0.07	0.098
	Well-controlled T2D	0.22	1	1.000
	Poorly-controlled T2D	<0.0001	1	1.000
Well-controlled T2D	Control	<0.0001	0.777	0.184
	Pre-diabetes	0.22	1	1.000
	Poorly-controlled T2D	0.157	1	1.000
Poorly-controlled T2D	Control	<0.0001	0.008	0.001
	Pre-diabetes	<0.0001	1	1.000
	Well-controlled T2D	0.157	1	1.000

*Table shows post-hoc analysis comparing HN, MOTS-c, and ADP between control, prediabetes, well-controlled T2D and in poorly-controlled T2D subjects*.

### Correlations of MDP and ADP With Biochemical and Metabolic Parameters

**Figure 2:**
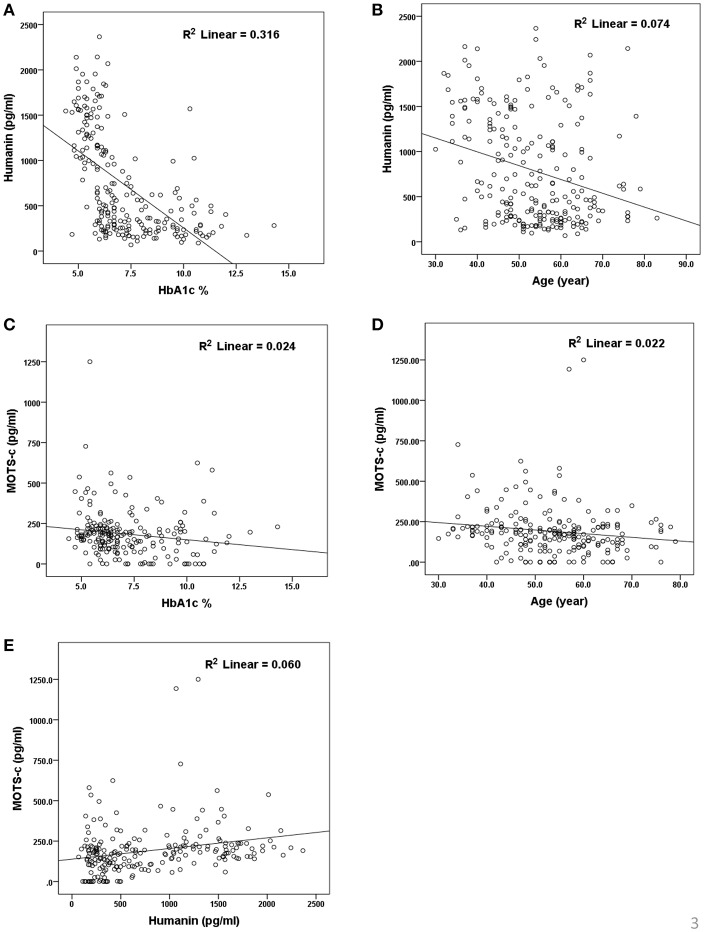
Association of serum HN **(A,B)** and MOTS-c **(C,D)** with HbA1C, age and between HN and MOTS-c **(E)**. HbA1C, glycated hemoglobin. The “*R*^2^” values represent coefficient of determination in linear regression analysis.

The relationship of HN, MOTS-c, and ADP to age, lipids (TCH, LDL, and HDL triglycerides), liver function (AST) were analyzed by Spearman Rank correlation. HN was positively correlated with TCH (*r* = 0.158; *P* = 0.017), HDL (*r* = 0.172; *P* = 0.009), LDL (*r* = 0.212; *P* < 0.001), and insulin sensitivity marker ADP (*r* = 0.213; *P* < 0.003). And negatively correlated with HbA1c (*r* = −0.657; *P* < 0.0001), glucose (*r* = −0.577; *P* < 0.0001), ALT (*r* = −0.187; *P* = 0.004), TG/HDL (*r* = −0.234; *P* < 0.0001), and age (*r* = −0.256; *P* < 0.0001) (Table 2A). MOTS-c was positively correlated with BMI (*r* = 0.142; *P* = 0.035), TCH (*r* = 0.253; *P* < 0.0001), LDL (*r* = 0.231; *P* < 0.001) and negatively correlated with HbA1c (*r* = −0.233; *P* = 0.001), glucose (*r* = −0.213; *P* = 0.002), and age (*r* = −0.208; *P* = 0.002) (Table 2C). HN and MOTS-c were positively correlated with each other (*r* = 0.312; *P* < 0.0001). To test the effect of those variables that showed significant association with HN and MOTS-c in bivariate analysis, these parameters were tested by linear regression analysis model to determine if any of the covariates could individually predict variations in HN and MOTS-c. The combined group multiple linear regression analysis showed that HbA1c (regression coefficient β = −0.442; *P* < 0.0001) and ALT (β = −0.127; *P* = 0.026) were the two independent predictors that were able to predict serum HN for combined group analysis) (Table 2B). Similarly, for MOTS-c linear regression analysis showed TCH as the only predicting factor in combined group analysis) (Table 2D). Association of HN with HbA1c (*R*^2^ = 0.316) and age (*R*^2^ = 0.074) and association of MOTS-c with HbA1c (*R*^2^ = 0.024) and age (*R*^2^ = 0.022) is shown in Figure 2. ADP levels were positively correlated with HN (*r* = 0.213; *P* = 0.003), age (*r* = −0.156; *P* < 0.029), and HDL (*r* = 0.292; *P* = 0.0001) and negatively correlated with HbA1c (*r* = −0.237; *P* < 0.001), weight (*r* = −0.153; *P* < 0.032), TG (*r* = −0.324; *P* < 0.0001), ALT (*r* = −0.319; *P* < 0.0001), TG/HDL (*r* = −0.374; *P* < 0.0001), and TCH/HDL (*r* = −0.254; *P* < 0.0001) (Table 2E). Multiple linear regression analysis showed HbA1c and weight to independently correlate with ADP in combined group analysis (Table 2F).

**Table 2A:** Spearman Rank correlation bivariate analysis of variables associated with HN.

**Combined group analysis**		
	***r***	***p***
MOTS-c (pg/ml)	0.312^**^	<0.0001
ADP (μg/ml)	0.213^**^	0.003
Age	−0.256^**^	<0.0001
Height (m)	0.095	0.144
Weight (kg)	0.054	0.401
BMI (Kg/m^2^)	−0.019	0.763
SBP (mm Hg)	−0.020	0.841
DBP (mm Hg)	0.047	0.634
HbA1C %	−0.657^**^	<0.0001
TCH (mmol/L)	0.158^*^	0.017
TG (mmol/L)	−0.195^**^	0.003
HDL (mmol/L)	0.172^**^	0.009
LDL (mmol/L)	0.212^**^	0.001
ALT (U/L)	−0.187^**^	0.004
TG/HDL	−0.234^**^	0.000

* *Significance 2-tailed correlation significance of greater than 0.05*.

** *Significance 2-tailed correlation significance of greater than 0.01*.

**Table 2B:** Multiple linear regression analysis of variables that showed significant association with HN.

**Combined group analysis**		
	**β**	***P***
MOTSc (pg/ml)	0.246	<0.0001
ADP (μg/ml)	0.046	0.514
Age	−0.109	0.063
HbA1C	−0.442	0.0001
TCH	0.359	0.427
Triglycerides	−0.321	0.244
HDL	−0.107	0.449
LDL	−0.186	0.639
ALT	−0.127	0.026
TG/HDL	0.129	0.572

**Table 2C:** Spearman Rank correlation bivariate analysis of variables associated with MOTS-c.

**Combined group analysis**		
	***r***	***p***
HN (pg/ml)	0.312^**^	<0.0001
ADP (μg/ml)	−0.0285	0.699
Age	−0.208^**^	0.002
Height (m)	0.021	0.755
Weight (kg)	0.120	0.075
BMI (Kg/m^2^)	0.142^*^	0.035
SBP (mm Hg)	0.025	0.811
DBP (mm Hg)	0.018	0.864
HbA1C %	−0.233^**^	0.001
TCH (mmol/L)	0.253^**^	0.000
TG (mmol/L)	0.025	0.724
HDL (mmol/L)	0.118	0.090
LDL (mmol/L)	0.231^**^	0.001
ALT (U/L)	0.008	0.904
TG/HDL	−0.025	0.723
TCH/HDL	0.111	0.108

* *Significance 2-tailed correlation significance of greater than 0.05*.

** *Significance 2-tailed correlation significance of greater than 0.01*.

**Table 2D:** Multiple linear regression analysis of variables that showed significant association with MOTS-c.

**Combined group analysis**		
	**β**	***P***
HN (pg/ml)	0.246	<0.0001
Age	−0.121	0.104
BMI (Kg/m^2^)	0.034	0.635
HbA1C %	−0.093	0.382
TCH (mmol/L)	0.386	0.037
LDL (mmol/L)	−0.255	0.166
ALT (U/L)	0.006	0.938

**Table 2E:** Spearman Rank correlation bivariate analysis of variables associated with ADP.

**Combined group analysis**		
	***r***	***p***
MOTS-c (pg/ml)	−0.029	0.699
Humanin (pg/ml)	0.213^**^	0.003
Height (m)	−0.139	0.051
Weight (kg)	−0.153^*^	0.032
BMI (Kg/m^2^)	−0.106	0.140
SBP (mm Hg)	−0.028	0.793
DBP (mm Hg)	−0.070	0.514
HbA1C %	−0.237^**^	0.001
TCH (mmol/L)	0.003	0.968
TG (mmol/L)	−0.324^**^	<0.0001
HDL (mmol/L)	0.292^**^	<0.0001
LDL (mmol/L)	0.039	0.597
ALT (U/L)	−0.319^**^	<0.0001
TG/HDL	−0.374^**^	<0.0001
TCH/HDL	−0.254^**^	<0.0001

* *Significance 2-tailed correlation significance of greater than 0.05*.

** *Significance 2-tailed correlation significance of greater than 0.01*.

**Table 2F:** Multiple linear regression analysis of variables that showed significant association with ADP.

**Combined group analysis**		
	**β**	***P***
HN (pg/ml)	0.088	0.314
Weight (kg)	−0.162	0.042
HbA1C %	−0.231	0.034
TG (mmol/L)	0.057	0.834
HDL (mmol/L)	0.067	0.564
ALT (U/L)	−0.116	0.113
TG/HDL	−0.168	0.571
TCH/HDL	−0.044	0.658

*Spearman Rank correlation analysis was used for calculation of associations between HN (2A), MOTS-c (2C), ADP (2E), and clinical variables. If individual bivariate correlations achieved statistical significance those variables were included for multivariate linear regression analysis to test the joint effect of these parameters on HN (2B), MOTS-c (2D), and ADP (2F). Age, weight, HbA1c, glycated hemoglobin; MOTS-c, mitochondrial open reading frame of the 12S rRNA type-c; HN, humanin; ADP, adiponectin; BMI, body mass index; SBP, systolic blood pressure; DBP, diastolic blood pressure; ALT, alanine aminotransferase; TCH, total cholesterol; HDL, high density lipoprotein; LDL, low density lipoprotein; VLDL, very low density lipoprotein; TG, triglycerides; R, Spearman rank correlation coefficient; β, standardized linear regression beta coefficients; P < 0.05 was considered as significant*.

**Table 3:** Effects of antidiabetic medications on serum HN, MOTS-c and ADP levels.

**Diabetes Treatment**	***N =***	**HN (pg/ml) Mean ± SD**	**MOT-c (pg/ml) Mean ± SD**	**ADP (μg/ml) Mean ± SD**	***p***
Newly diagnosed diabetes (diet and lifestyle?)	11	431.1 ± 203.7	172.3 ± 89.9	7.6 ± 3.5	
Metformin	32	470.1 ±256.4	162.9 ± 107.6	7.9 ± 5.2	NS
Insulin	24	508.9 ± 343.1	156.8 ± 85.9	9.9 ± 6.7	NS
Any oral hypoglycemic agents + metformin	30	341.2 ± 219.4	170.7 ± 111.0	7.3 ± 7.2	NS
Insulin + any oral hypoglycemic agents	27	432.8 ± 203.7	202.7 ± 140.9	6.6 ± 4.1	NS

*Effects of hypoglycemic agents on circulating HN, MOTS-c, and ADP. Data are represented as Mean ±SD. Group comparision was done by one-way ANOVA analysis with post-hoc Bonferroni correction*.

## Discussion

Our study demonstrates that the circulating MDPs (HN and MOT-c), and ADP levels were reduced in T2D and significantly related to HbA1c. We found that HN levels were decreased in patients with prediabetes, a condition that carries an annualized progression rate to diabetes of 5–10% (31). HN was further decreased in T2D and may be influenced by the degree of glycemic control. Mean HN concentrations were reduced by 62% in the prediabetes group, 66 % in diabetes subjects with good control (HbA1c <7%) and 77% in poorly controlled diabetes subjects (HbA1c >7%) compared to non-diabetes subjects. In contrast, mean MOTS-c concentrations were reduced by 27% in diabetic subjects with a range of HbA1c between 7.6 and 10% compared to non-diabetic controls.

HN levels, as expected, were negatively associated with other clinical markers of insulin resistance, triglyceride and ALT however only ALT and HbA1c independently contributed to the variation in the circulating HN levels in our combined cohort. In the diabetic groups, an association with BMI was identified in the poorly-controlled T2D group suggesting an added negative impact of obesity. HN concentrations have positive correlation with TCH, LDL, and HDL and negative correlation with triglycerides which cannot be fully explained in this study. However, previous studies in obese mice show intracerebroventricular infusion of HN analog decreases hepatic triglyceride accumulation promotes hepatic microsomal triglyceride transfer protein (MTTP) activity and there by increases serum triglycerides (32).

Serum HN concentration inversely correlated with age in the combined group, since the T2D group with poor control was significantly older than the non-diabetic controls and since HN is lower in T2D than non DM subjects we performed multiple linear regression analysis comparisons and found that age is not a significant contributor in the multivariable model. A previous study plagued by the same confounder reported that circulating HN to be lower in elderly people and in prediabetes subjects (33); and is confirmed in our study. Altered HN levels in prediabetes and T2D could serve as a potential biomarker; however, additional work is required to determine the contribution of age and other factors that are modifiable such as fitness level and adiposity. HN levels are reported to be altered in other metabolic diseases including type 1 diabetes (34), cardiovascular disease (35, 36), memory loss, amyotrophic lateral sclerosis (37), stroke (38), and inflammation (39). HN is present in the blood, cerebrospinal fluid and in seminal fluid (40–42), however, it is still not clear which tissue(s) contributes most to the circulating HN pool in humans. Given its mitochondrial origin, one would presume majority of the metabolically active tissues such as heart, liver, adipose tissue, and skeletal muscle would contribute to the circulating HN pool.

Exercise promotes skeletal muscle HN expression in prediabetes subjects, suggesting a potential role for HN in glucose metabolism (43). In rodents, HN and its analogs promote insulin sensitivity and restore glucose homeostasis under hyperinsulinemic euglycemic clamp conditions (23). Peripheral administration of HN delayed the onset of diabetes and improved beta cell survival in non-obese type 1 diabetes (NOD) mice (26). In cultured beta cells, HNG-F6A, a potent analog of HN, promoted mitochondrial metabolism and ATP generation, indicating a role for HN in substrate metabolism (22). HNG-F6A reduces oxidative stress and prevents atherosclerosis progression in ApoE knockout mice (44). The above studies suggest that HN and its analog may function as potent regulators of oxidative stress and glucose metabolism.

Accumulating literatures suggest that mitochondrial dysfunction is a salient feature in T2D. Reduction in skeletal muscle mtDNA and diminished oxidative capacity in T2D subjects have been observed in T2D subjects who fail to respond to exercise intervention (45), decreased mtDNA content strongly correlates with IR and lower mtDNA strongly associated with reduced capacity of glucose-stimulated insulin secretion in prediabetes and T2D subjects (46).

The lower levels of HN in T2D subjects might be a net effect or secondary response to the elevated ROS production, oxidative stress and lowered mtDNA copy number (47), which are all risk factors for IR. Preceding studies have shown no significant changes in HN levels between healthy men and women, and this was confirmed here (34). HN levels were not affected by the differing anti-diabetes treatment regimens: HN was similar in newly diagnosed T2D subjects and those on anti-diabetes therapy for more than 6 months. Anti-diabetes therapy such as insulin, metformin, combination of insulin + an oral hypoglycemic agent or combination of metformin + an oral hypoglycemic agent for more than 6 months did not alter serum HN levels in our study cohort.

Increasing adiponectin expression reduces metabolic abnormalities associated with obesity and T2D. Our data are in agreement with a previous report (48) that ADP concentrations are reduced in prediabetes and T2D. Our data are also consistent with the findings that ADP knockout mice have decreased mitochondrial content in skeletal muscle associated with insulin resistance and mitochondrial dysfunctional skeletal muscle (49): and that adiponectin may affect mitochondrial biogenesis (30). Thus the lower mitochondrial function may be a consequence of the lower adiponectin levels: certainly the association of ADP with HN was clearly seen in this study.

Serum MOTS-c levels reflected those of HN, although only in poorly-controlled T2D subjects compared to non-diabetes subjects. The T2D poor control group was significantly older compared to control group, however, age was found inversely correlate with MOTS-c concentrations in bivariate analysis but not in multivariate modeling. As in the case of HN, we are not able to determine the exact contribution of poorer glycemic control in T2D or aging on serum MOTS-c in these patients. MOTS-c levels in the circulation were positively correlated with lipids TCH, LDL, and HN in the whole cohort in bivariate analysis but only HN and TCH remained significant in multiple linear regression analysis. MOTS-c negatively correlated with age and HbA1c in combined group analysis.

There is little known about MOTS-c in metabolic disease (38, 39), although preclinical studies demonstrate a positive impact on glucose metabolism. Recently, two studies reported conflicting findings in circulating MOTS-c levels in lean and obese subjects. Cataldo et al. reported MOTS-c levels to be the same in a small group of obese and non-obese subjects with a positive association with IR only in lean subjects (50). Conversely, Du et al. reported that MOTS-c levels are significantly lower in obese male children and adolescents compared to females, and also showed a negative correlation with BMI, HOMA, HbA1c, and fasting insulin in male subjects (51). Of note, the study from Du et al. used commercially available elisa kit (# CEX132HU, Cloud clone corporation, China) and reported circulating MOTS-c to be nearly 1,000-fold higher compared to the study from Cataldo et al. which employed non-commercial elisa kit. This suggests that the immunoreactive species in the circulation detected by these two different kits are not same. The favorable effects of MOTS-c on insulin sensitivity and glucose metabolism have been seen in animal models (24), with MOTS-c enhancing glucose utilization, promotion of insulin sensitivity and restoration of metabolic homeostasis through activation of an AMPK and AKT dependent mechanism in rodent skeletal muscle and *in vitro* models. MOTS-c protects mice against insulin resistance induced by obesity and aging (24). MOTS-c inhibits bone loss and reduced the osteoporosis rate in an ovariectomy-induced osteoporosis mice model (52), promotes endothelial function in rodents and the level was reduced in patients with impaired endothelial function (53).

HN levels correlated positively with MOTS-c and with ADP in our group analysis both in univariate and multivariate modeling. One possible explanation for this positive relationship between MDPS could be that both HN and MOTS-c are released simultaneously into circulation to counter act the oxidative stress induced by hyperglycemia. This is supported by previous findings in cellular and rodent models that HN inhibits oxidative stress, rescues mitochondrial function, lowers apoptotic rate and enhances glucose metabolism (54). It has been shown that HN analog (HNG) enhances intracellular anti-oxidant capacity, preserves mitochondrial membrane potential, ATP levels and restores mitochondrial integrity in rat myoblasts (H9c2 cells) (55), inhibits mitochondrial complex 1 activity and prevents mitochondrial dysfunction and oxidative stress induced by H_2_O_2_ in isolated cardiac mitochondria (56). MOTS-c is also regulated by stress; it translocates to the nucleus in response to glucose restriction-induced metabolic stress and modulates nuclear gene expression in AMPK dependent manner (57).

The strength of this study is being a relatively large scale cohort conducted to address the importance of MDP in diabetes and non-diabetic control subjects. Limitations of the study include its retrospective design dependent on previously measured clinical parameters. Both T2D patients and the control group were overweight or obese; therefore it was not possible to determine the contribution of obesity to circulating MDP. Samples used in the study were randomly collected for this reason it was not possible to determine HOMA-IR or other indices of insulin resistance derived from fasting glucose and insulin levels. Furthermore, the non- diabetes subjects were younger obscuring the contribution of age on MDP concentrations.

In conclusion, the present study demonstrates the MDPs HN and MOT-c expression were lower in T2D and were related to the HbA1c, and HN was associated with ADP, giving additional evidence that mitochondrial dysfunction contributes to glycemic dysregulation and metabolic effects in T2D. Additional studies are required to determine the role of MDPs in the metabolic dysregulation of T2D.

## Ethics Statement

This was a cross-sectional study performed using blood samples collected from Qatari T2D subjects attending the outpatient department and endocrinology clinics at Hamad Medical Corporation according to an IRB approved protocol (HMC#9093/09 and WCM-Q#13-00063). All study participants gave informed written consent before participation in the study.

## Author Contributions

MR designed the experiments, performed the experiments, analyzed, interpreted data, and prepared the manuscript. IB performed ELISA studies with subsequent analysis. JJ performed ELISA studies prepared graphical presentation. PC analyzed the data and performed statistical analysis. CA provided the samples and revised the manuscript. MS verified all obtained results, analyzed the data, and revised the manuscript. SA and A-BA-S designed the experiments, analyzed data, and revised the manuscript. All authors took part in preparation and modification of figures and the manuscript.

### Conflict of Interest Statement

The authors declare that the research was conducted in the absence of any commercial or financial relationships that could be construed as a potential conflict of interest.

## Abbreviations

T2D: Type 2 diabetes
IR: insulin resistance
MOTS-c: mitochondrial open reading frame of the 12S rRNA type-c
HN: humanin
BMI: body mass index
SBP: systolic blood pressure
DBP: diastolic blood pressure
TCH: total cholesterol
HDL: high density lipoprotein
LDL: low density lipoprotein
TG: triglycerides
HbA1c: glycated hemoglobin
ALT: alanine aminotransferase
TCH: total cholesterol
AMPK: 5’ AMP-activated protein kinase
ROS: reactive oxygen species
H_2_O_2_: hydrogen peroxide.
